# Increasing psychopharmacology clinical trial success rates with digital measures and biomarkers: Future methods

**DOI:** 10.1038/s44277-024-00008-7

**Published:** 2024-05-24

**Authors:** Jacob E. Reiter, Stefanie Nickels, Benjamin W. Nelson, Erin Rainaldi, Lily Peng, P. Murali Doraiswamy, Ritu Kapur, Amy Abernethy, Andrew Trister

**Affiliations:** 1 Verily Life Sciences LLC, South San Francisco, CA USA; 2 Department of Psychiatry, Harvard Medical School and Beth Israel Deaconess Medical Center, Boston, MA USA; 3 Department of Psychiatry and Behavioral Sciences, Duke University School of Medicine, Durham, NC USA; 4 Duke Institute for Brain Sciences, Duke University School of Medicine, Durham, NC USA; 5 Department of Neurology, Radboud University Medical Center, Nijmegen, Netherlands

**Keywords:** Predictive markers, Psychiatric disorders, Clinical trial design, Biomarkers, Depression

## Abstract

Psychiatric trials have some of the lowest success rates across therapeutic areas, resulting in decreased investment in psychopharmacological drug development even as the need for more effective treatments grows. Digital measures and digital biomarkers (DBMs) provide one potential avenue for ameliorating three of the largest problems impeding clinical trial success in psychiatry: diagnostic heterogeneity, endpoint subjectivity, and high placebo response rates. First, DBMs may address heterogeneity and comorbidity in psychiatric nosology by identifying predictive DBMs of treatment response via the targeting of drugs to psychiatric subtypes. Second, DBMs can provide objective measures of physiology and behavior that when grounded in meaningful aspects of health (MAH) could support use for regulatory decision-making. By objectively and continuously measuring aspects of a patient’s disease that the patient wants to improve or prevent from getting worse, DBMs might provide clinical trial endpoints that are more sensitive to treatment effects as compared to traditional clinician-reported outcomes. Lastly, DBMs could help address challenges surrounding high placebo response rates. Development of predictive DBMs of placebo response may allow for improved enrichment study designs to reduce placebo response. Objective digital measures may also be more robust against the placebo effect and offer an improved study endpoint alternative. Successful deployment of DBMs to address the historical challenges facing psychiatric drug trials will require close collaboration between industry, academic, and regulatory partners.

## Lay summary

Psychiatric clinical trials fail at high rates, leading to less investment in new treatments despite the growing need for new innovations. Digital measures, such as data from wearable devices, offer a potential solution to core challenges in psychiatric trials. They could identify specific patient groups who may respond better to certain drugs, provide objective measurements of symptoms, and help resolve the impact of the placebo effect on trials.

## Introduction

Patients with psychiatric disorders are in need of new treatments. Despite advances in research, pharmacologic treatment remission rates are low, and real-world efficacy is often questioned [1]. An estimated 70% of major depressive disorder (MDD) patients will not remit from first-line antidepressants, and approximately 30% of MDD [2] and schizophrenia [3] cases are treatment-resistant. Furthermore, the prevalence of any mental illness and serious mental illness has increased by approximately 20% and 50%, respectively, over the past 12 years [4].

Pharmaceutical and biotechnology investment has resulted in more molecules identified as potential therapies, however only 6.2% of the compounds that enter clinical development ultimately receive regulatory approval [5]. Psychiatric clinical trials are amongst the least likely to succeed today. Across the 21 largest therapeutic areas, psychiatry has the lowest probability of success in both Phase I and Phase II, and the third lowest probability of Phase III success [5].

Even in the face of dire medical need, the apparent risk inherent to clinical development in psychiatry seems to have had a deterrent effect, coinciding with a considerable exodus from psychiatric investment. The National Institute of Mental Health’s (NIMH) funding of clinical trials in schizophrenia, bipolar disorder, and MDD decreased by 90% between 2016–2019 [6] and commercial psychopharmacological research has seen an estimated 70% spending reduction [7, 8].

Amidst declining success rates and constrained investment, there is a measured recognition within psychiatric research of the need for pragmatic innovation. Here we explore the integration of digital measures into psychiatric trials to address three central problems impeding clinical trial success: disease heterogeneity, imprecise and subjective measurement, and high placebo response.

## Digital measures in psychiatry

Digital measure is a broad term used to describe any measurement collected using digital technology. More narrowly, digital biomarkers (DBMs) are digitally measured “indicator[s] of normal biological processes, pathogenic processes, or biological responses to an exposure or intervention, including therapeutic interventions” [9].

Digital measures, particularly those obtained via everyday-wearable sensors, allow for continuous and unobtrusive health-related data collection in real-world settings. These include measures of physiology (e.g., pulse rate via photoplethysmography) and behavior (e.g., insomnia/hypersomnia and psychomotor agitation/retardation via actigraphy) that have the potential to circumvent the subjectiveness of self-report or clinician rating. These measures could reflect daily-living with increased granularity, providing an ideal platform for disease assessments grounded in meaningful aspects of health (MAH).

Considerable literature has investigated the use of digital measures and DBMs in behavioral health [10] and we postulate that these digital health technologies (DHT) have the potential to accelerate psychiatric innovation and drug development and discovery. In the following sections, we describe how digital measures can be leveraged to address core clinical and regulatory hurdles by improving precision in matching therapies to patients, objectively measuring impact, and addressing the limitations of placebo-controlled studies when placebo effects are high.

## 1. Heterogeneity and comorbidity of DSM disorders

### The problem

**Fig. 1:**
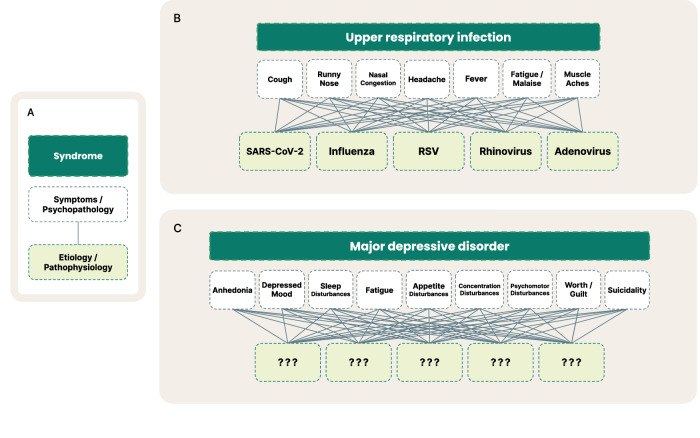
**Visualized comparison of the current syndrome-level framework of DSM psychiatric disorders to other diseases**. **A** is an overview of the breakdown from broader syndrome level (*dark green*), to symptoms and psychopathology (*white*), to underlying etiology and pathophysiology (*light green*). **B** depicts some of the many known underlying viruses that manifest as often identical symptoms, which at first may be called an upper respiratory infection until a test can verify the specific virus or cause. **C** depicts the analogous comparison in psychiatry with the example of MDD. Here, the syndrome of MDD is composed of the 9 core symptoms laid out by the DSM, for which there may exist many underlying etiologies that today are not well-understood.

One believed reason for high clinical trial failure rates is that psychiatric disorders are highly heterogeneous and comorbid. For example, there are over 1,000 unique MDD symptom profiles [11] and over 70% of patients with MDD also meet diagnostic criteria for another psychiatric disorder [12]. As a result, diagnostic systems, such as the Diagnostic and Statistical Manual of Mental Disorders (DSM-5), suffer from imprecise psychiatric nosology as diagnostic categories share transdiagnostic symptoms, resulting in low test-retest diagnostic reliability (e.g., MDD kappa = 0.28) [13]. Unlike most other specialties, psychiatry must rely on criteria-defined syndromes, as the underlying pathophysiologies are not well understood (Fig. 1). Notable recent efforts to address these challenges include the Research Domain Criteria (RDoC) [14], a framework for researchers to align pathophysiology to domains and constructs of illness, and the Hierarchical Taxonomy of Psychopathology (HiTOP), an alternative classification system to the DSM [15].

**Fig. 2:**
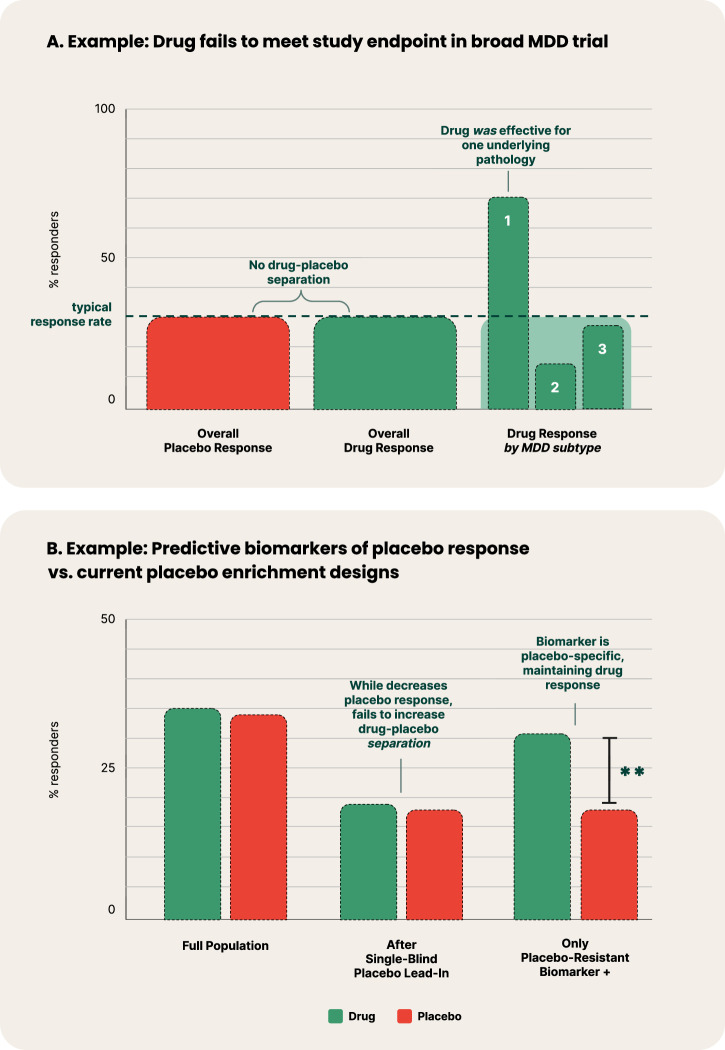
**Examples of predictive biomarkers of treatment and placebo response**. **A** Hypothetical example where treatment is highly effective in a specific pathophysiological transdiagnostic syndrome profile of MDD (labeled as ‘1’), yet fails to meet its study endpoint in a placebo-controlled trial that recruits MDD broadly when looking at overall drug-placebo separation. **B** Comparison between hypothetical implementation of a single-blind placebo lead-in and a predictive biomarker of placebo response. The example of the single-blind placebo lead-in depicts the common trend in which the successful decreasing of placebo response also decreases drug response, subsequently failing to increase drug-placebo separation. Conversely, to be a predictive biomarker of placebo response, the measure’s prediction must be specific to placebo response, and neutral or minimally specific to drug response. In this example, the placebo-resistant biomarker + population has reduced placebo response, with only minimally reduced drug response, thereby demonstrating drug-placebo separation.

Diagnostic heterogeneity and lack of specificity undermine drug development in psychiatry [16]. When a novel drug is developed for a specific heterogeneous diagnosis, the mechanisms of action may only be therapeutic for a subset of participants with a specific underlying pathophysiology. Therefore, a lack of significant treatment effects at the disease population level could be masking high effectiveness for a specific subtype (Fig. 2A).

### Solutions: Predictive DBMs and diagnostic subtyping

Recently, clinical progress in many therapeutic areas has come with shifts towards personalized medicine with predictive biomarkers [17], which identify patients who are more likely to experience a certain outcome in response to a *specific* treatment [18] (e.g. HER2-positive breast cancer patients respond more favorably to trastuzumab) [17]. Narrowing psychiatric populations to those predicted to have a greater likelihood of response to a specific treatment holds massive advantages. In a review of over 9500 clinical trials, programs that utilized predictive biomarkers had a three-fold higher likelihood of approval from Phase 1 (25.9% vs*.* 8.4%), as well as higher phase transition success rates at every phase, including from Phase II to Phase III (46.9% *vs* 28.8%) where psychiatric trials fail most [5].

Predictive biomarkers are of increasing interest in psychiatry as demonstrated by NIMH’s launch of a new precision psychiatry initiative, Individually Measured Phenotypes to Advance Computational Translation in Mental Health, which is attempting to identify predictive biomarkers from computerized behavioral tasks [19]. Incremental to behavioral tasks, digital measures from wearables, implantables, injectables, ambient sensors or smartphone sensors can objectively capture physiology and real-world behavior [18]. As such, they have the potential to advance our understanding of pathophysiological subtypes and subsequently function as predictive DBMs. Specific steps for the identification of predictive DBMs could include digital measures in a clinical trial to retrospectively identify candidate predictive DBMs, followed by validation thereof in subsequent clinical trials with a focused enrichment design with specific diagnostic subtypes. The selection of which digital measures to utilize should be guided both by hypotheses of the disease pathology and the drug mechanism of action.

As evidenced by the reduced investment in psychiatric drug development over the last decades, pharmaceutical companies are in need of methods to de-risk psychiatric programs. The inclusion of digital measures in clinical trials can function as a ‘fail-safe,’ where digital measures are integrated into ‘non-selection’ late-stage protocols, and could help extract scientific value from trials that fail, but enable the identification of biomarker-positive patients for follow-up research.

While predictive biomarkers have the potential to reduce the patient population eligible for a specific treatment due to greater specificity of patients that are likely to have a treatment effect, they also have the potential to broaden indications by expanding from a single DSM diagnosis to transdiagnostic trials enrolling multiple DSM disorders. This method has already seen success in one Phase II trial that included both MDD and post-traumatic stress disorder patients based on predictive biomarker status [20].

## 2. Endpoint mismatch and subjectivity

### The problem

Another challenge that is core to the high failure rates in psychiatric trials is the subjective and combinatorial measures used. As of today, outcome evaluation in psychiatry is typically based on broad clinician-reported outcomes (ClinROs) addressing the severity or existence of wide monolithic syndromes. However, even questionnaires and psychodiagnostic assessments completed by trained clinicians are challenged by subjectivity. For example, the Hamilton Depression Rating Scale (HAM-D), one of only two FDA-approved primary endpoints in MDD clinical trials, has consistently struggled to show adequate inter-rater and test-retest reliability across items [21].

In addition, when a specific etiology is hypothesized, or an investigational drug has a known neurophysiological effect, endpoints would ideally match specific corresponding physiological or behavioral outcomes. However, endpoints in psychiatric trials focus on sum-scores of broad scales. It is believed that these sum-scores may actually conceal or diffuse the response of a drug in a clinical trial [22, 23].

### Solutions: Digital measures grounded in meaningful aspects of health

Digital measures of physiology and behavior provide rich, textured, and continuous information about daily functioning, and have the potential to serve as an objective source for endpoints that could supplement the subjectiveness of patient-reported outcomes (PROs) or ClinROs [23]. Digital measures have repeatedly been shown to be capable of high test-retest reliability [24].

In selecting a digital measure for a clinical trial, it can be advantageous to employ one grounded in MAH, defined as aspects of a disease that a patient “(a) does not want to become worse, (b) wants to improve, or (c) wants to prevent” [25]. A drug trial might not find a statistically significant or clinically meaningful reduction in overall symptom severity with a ClinRO but might do so on a given MAH measure (e.g., decreased duration of panic attacks as measured by a wrist-worn device or decreased time spent at home for agoraphobia as measured by a smartphone). This scenario of discrepancy highlights the importance of selecting a measure based on validated MAH, such that demonstrating efficacy on the digital measure would be meaningful to patients and therefore useful in assessing the value of the drug. Digital measures, however, may be highly difficult, or even impossible, to develop for a wide variety of critical symptoms and MAH in psychiatry. As such digital measures need not replace PROs and ClinROs, but can rather supplement the evidence-generation process.

DHTs are starting to be leveraged by the FDA and industry as demonstrated by the FDA acceptance of digital endpoints in pulmonary fibrosis [26] and nocturnal scratch [27]. The FDA has also proposed Patient-Focused Drug Development guidance [28] that has been adopted for digital measures [25] and successfully implemented for Parkinson’s disease [29].

## 3. Placebo response

### The problem

The third and perhaps most significant factor driving trial failures in psychiatry is the high and growing rates of placebo response [7, 30]. One systematic review of 252 antidepressant trials found a consistent placebo response rate today that is now between 35% and 40% [31]. High and rising rates of placebo response are an alarming challenge for psychiatric trials and can make drug approvals increasingly challenging by raising the barrier to demonstrate efficacy. An FDA publication emphasized the magnitude of the problem, revealing, for example, that schizophrenia placebo response has increased with average reductions in Positive and Negative Syndrome Scale growing from −4.3 pre-2009 to −8.5 post-2009, while drug response has decreased from −9.0 pre-2009 to −3.4 post-2009 [32].

A number of trial designs have been employed for decades to reduce placebo response, including single-blind placebo lead-ins and delayed-start designs; yet, meta-analyses have found them to be ineffective [33]. More recent designs including the sequential parallel comparison design hope to have more success [34]. At the center of this challenge is that the methods explored to date which aim to reduce placebo response often also inadvertently reduce the treatment effect, thereby not increasing the drug-placebo separation (Fig. 2B) [35].

### Solutions: Predictive DBMs and objective digital measures

As discussed in **Heterogeneity and Comorbidity of DSM Disorders**, predictive DBMs of response to specific treatments have the potential to address heterogeneity in psychiatry, and by definition would also aim to increase drug-placebo separation. However, an even more targeted solution to the challenge of high placebo effect could be to similarly develop predictive biomarkers, but for a placebo response, instead of specific treatment response. A predictive biomarker of *placebo* response or resistance identifies patients who are more or less likely to respond to placebo, respectively, while remaining comparatively neutral in response to active treatment (Fig. 2B). This constitutes an important distinction from a prognostic biomarker, which identifies the likelihood of a clinical event (e.g. remission), but does not require demonstrating a difference between placebo and treatment response [18]. This method was recently used successfully to retrospectively classify patients as either placebo responders or nonresponders in a chronic pain trial [36].

A development of this type must also have clear value for patients, and not serve to only artificially inflate trial success rates. A predictive biomarker of placebo response can have similar practical applications to its treatment response counterpart in the clinic: allowing clinicians to know when to avoid unnecessary pharmacological intervention and side effects when alternative treatments can work.

Separately, the objectivity of digital measures used as trial endpoints as described in **Endpoint Mismatch and Subjectivity**, in particular physiological measures, have the potential to be less sensitive to placebo response. Modality-specific placebo effects have been established across clinical outcome assessments (COAs). For example, a meta-analysis of anxiety disorder trials found that ClinROs are more likely to present larger placebo effects than PROs [37], and a study of performance outcomes (PerfOs) in schizophrenia exhibited a low placebo effect size on symptom improvement [38]. Future literature might evaluate further the robustness of objective digital measures to the placebo effect.

## Limitations and barriers to digital measure adoption into clinical trials

Despite the potential of digital measures to address challenges in psychopharmacology clinical trials, limitations and barriers hinder their adoption. First, digital measures primarily assess behavioral (e.g., sleep dynamics) and physiological (e.g., cardiovascular functioning) manifestations of the underlying pathophysiology, limiting their ability to evaluate molecular and biological mechanisms that may serve as drug targets or core psychological constructs related to diagnostic criteria. Second, technological and regulatory obstacles pose significant challenges. Digital measures necessitate extensive analytical and clinical validation, a time-consuming and expensive process requiring high cross-sector expertise. Regulatory approval requires establishing a clear link between digital measures and MAH, a foundational step that remains incomplete in psychiatry. Moreover, the use of proprietary algorithms should not impede adoption as most regulatory-cleared 510(k) medical devices use proprietary algorithms and receive regulatory clearance if prespecified predicate accuracy thresholds are met.

## Conclusion

Psychiatric trials are in need of novel solutions to address core challenges disrupting the development of improved treatments. Digital measures allow for continuous, objective, ecologically valid, and unobtrusive capture of patient function, and with specific strategies may be a powerful means of reducing risk for psychopharmaceutical drug development. Despite this promise, limitations in digital measures’ coverage of pathophysiology as well as technological and regulatory hurdles have prevented their successful deployment into clinical trials.

Overall, digital measures may allow for (1) addressing diagnostic heterogeneity through the identification of diagnostic subtypes and the development of predictive biomarkers of treatment response; (2) the development of DBMs representing MAH that can be used as endpoints for regulatory decision-making, and (3) development of predictive biomarkers of placebo response to improve enrichment study designs and reduce high placebo response.

### Citation diversity statement

The authors have attested that they made efforts to be mindful of diversity in selecting the citations used in this article.
